# The effector of Hippo signaling, Taz, is required for formation of the micropyle and fertilization in zebrafish

**DOI:** 10.1371/journal.pgen.1007408

**Published:** 2019-01-04

**Authors:** Xiaogui Yi, Jia Yu, Chao Ma, Guoping Dong, Wenpeng Shi, Hongtao Li, Li Li, Lingfei Luo, Karuna Sampath, Hua Ruan, Honghui Huang

**Affiliations:** 1 Key Laboratory of Freshwater Fish Reproduction and Development, Ministry of Education, State Key Laboratory Breeding Base of Eco-Environments and Bio-Resources of the Three Gorges Reservoir Region, School of Life Sciences, Southwest University, Beibei, Chongqing, China; 2 Cell & Developmental Biology Unit, Division of Biomedical Sciences, Warwick Medical School, University of Warwick, Coventry, United Kingdom; Georg-August Universitaet Goettingen Universitätsmedizin Göttingen, GERMANY

## Abstract

The mechanisms that ensure fertilization of egg by a sperm are not fully understood. In all teleosts, a channel called the ‘micropyle’ is the only route of entry for sperm to enter and fertilize the egg. The micropyle forms by penetration of the vitelline envelope by a single specialized follicle cell, the micropylar cell. The mechanisms underlying micropylar cell specification and micropyle formation are poorly understood. Here, we show that an effector of the Hippo signaling pathway, the Transcriptional co-activator with a PDZ-binding domain (Taz), plays crucial roles in micropyle formation and fertilization in zebrafish (*Danio rerio*). Genome editing mutants affecting *taz* can grow to adults. However, eggs from homozygous *taz* females are not fertilized even though oocytes in mutant females are histologically normal with intact animal-vegetal polarity, complete meiosis and proper ovulation. We find that *taz* mutant eggs have no micropyle. Taz protein is specifically enriched in mid-oogenesis in the micropylar cell located at the animal pole of wild type oocyte, where it might regulate the cytoskeleton. Taz protein and micropylar cells are not detected in *taz* mutant ovaries. Our work identifies a novel role for the Hippo/Taz pathway in micropylar cell specification in zebrafish, and uncovers the molecular basis of micropyle formation in teleosts.

## Author summary

In many fish, sperm enters eggs through a specialized channel called the ‘micropyle’. The micropyle is formed by a special follicle cell, the ‘micropylar cell’, which sits on the top of the developing egg during oogenesis, and forms the sperm entry canal. The underlying mechanisms of this process are unknown. We find that Taz, an effector of an important signaling pathway, the Hippo pathway, is specifically enriched in micropylar cells in zebrafish, and regulates formation of the micropyle. Loss of Taz function in females results in no micropylar cells, failure to form a micropyle on eggs, which are consequently, not fertilized. Our study identifies a new role for the Hippo/Taz pathway in cell fate specification in the ovary, and reveals a potential mechanism for forming the sperm entry port. Similar mechanisms might operate in other fish as well.

## Introduction

In vertebrates, fertilization occurs by two major strategies. Amniotes such as reptiles, birds and mammals, undergo copulation and internal insemination to ensure gamete fusion. The acrosome reaction is necessary for sperm to penetrate the zona pellucida, a protective egg envelope, and entry of sperm can occur at any position in the egg surface [1–3]. By contrast, most teleosts (bony fish) undergo external fertilization. Without a recognizable acrosome reaction, sperm entry in teleosts relies entirely upon a specialized funnel-like structure, the micropyle, in the chorion, an acellular coat of the egg [4–6]. Morphological and physiological studies of the micropyle in a variety of different teleost species suggest that channel formation results from the transformation of a special micropylar cell in mid-oogenesis [7–12]. The micropylar cell is morphologically distinct from other follicle cells surrounding the oocyte. Positioned over the oocyte animal pole, the micropylar cell is bigger in size and appears like an inverted cone in shape, in contrast to the flattened appearance of follicle cells, sometimes called ‘mushroom’-like [11–13]. The unique shape of the micropylar cell is gradually achieved during oogenesis. A cytoplasmic extension from the micropylar cell expands and extends through the developing vitelline envelope, till the extension tip contacts the oocyte membrane, as the vitelline envelope grows and perforation proceeds, the cytoplasmic extension becomes slim and long [14]. Finally, the micropylar cell degenerates, leaving a narrow canal called the ‘micropyle’ between the chorion and the egg [15, 16]. Previous studies in other teleosts revealed potential drilling forces of the micropylar cell. The aggregation and elongation of microtubules and tonofilaments in the cytoplasmic bulge of the micropylar cell provides internal forces [11, 14], and two opposing rotations between the oocyte and the covering follicle cell layer are thought to provide the external force for the micropylar cell to bore through the chorion [13, 17, 18]. Although these studies described the morphological process of micropyle formation, little is known about the molecular mechanisms underlying formation of this essential structure. A key line of evidence comes from studies of a zebrafish maternal-effect mutant *bucky ball* (*buc*), in which oocyte polarity fails to be established. This mutant has multiple micropyles in each egg, arising from the expanded animal identity in *buc* mutant oocytes [19, 20].

Hippo signaling plays a variety of roles in development, regeneration, tissue homeostasis, and stress response [21, 22]. The WW domain-containing transcription regulator protein 1 (Wwtr1) is a transcriptional co-activator with a PDZ-binding domain (Taz). Taz, together with Yes-associated protein (Yap), are downstream effectors of Hippo signaling. As a transcriptional co-activator, Taz usually exerts its functions by binding to transcription factors, such as Teads and Smad2/3, which modulates transcription of downstream genes [23]. As an oncoprotein, TAZ has been found up-regulated in many kinds of human cancers. TAZ also promotes epithelial-mesenchymal transition (EMT), migration and invasion of cancer cells, where cell morphology is altered and cytoskeleton is inevitably rearranged [24]. Yap/Taz can regulate cytoskeleton dynamics. In medaka (*Oryzias latipes*), Yap regulates cortical actomyosin activity and tissue tension by the downstream Rho GTPase activating protein ARHGAP, and mutants affecting the inhibitor of F-actin polymerization, *hirame*/*yap*, display reduced cortical actomyosin tension and a collapsed body shape [25]. Interestingly, similar to medaka *hirame*, the establishment of posterior body shape is disrupted in zebrafish *yap1;* *taz* double mutants [26, 27], which suggests that Yap1/Taz regulates cytoskeleton dynamics. In turn, Yap/Taz can be activated by environmental mechanical signals, for example matrix rigidity, which are usually transduced by the cytoskeleton [28, 29].

Tumors affecting two female organs, breast and ovaries, have been used extensively to study TAZ functions [30, 31]. However, to date, the role of TAZ in normal oogenesis and ovary differentiation has not been investigated. In a study of Taz function in zebrafish, we have unexpectedly found that Taz is required for the formation of micropyle during oogenesis. We show that *taz* transcripts are expressed maternally. When *taz* is knocked out, some homozygous *taz* mutants can survive to adulthood, and mutant females produce eggs with no micropyle. Our results suggest that Taz might regulate micropylar cell specification and morphogenesis during zebrafish oogenesis.

## Results

### Maternal Taz function is required for fertilization

**Fig 1:**
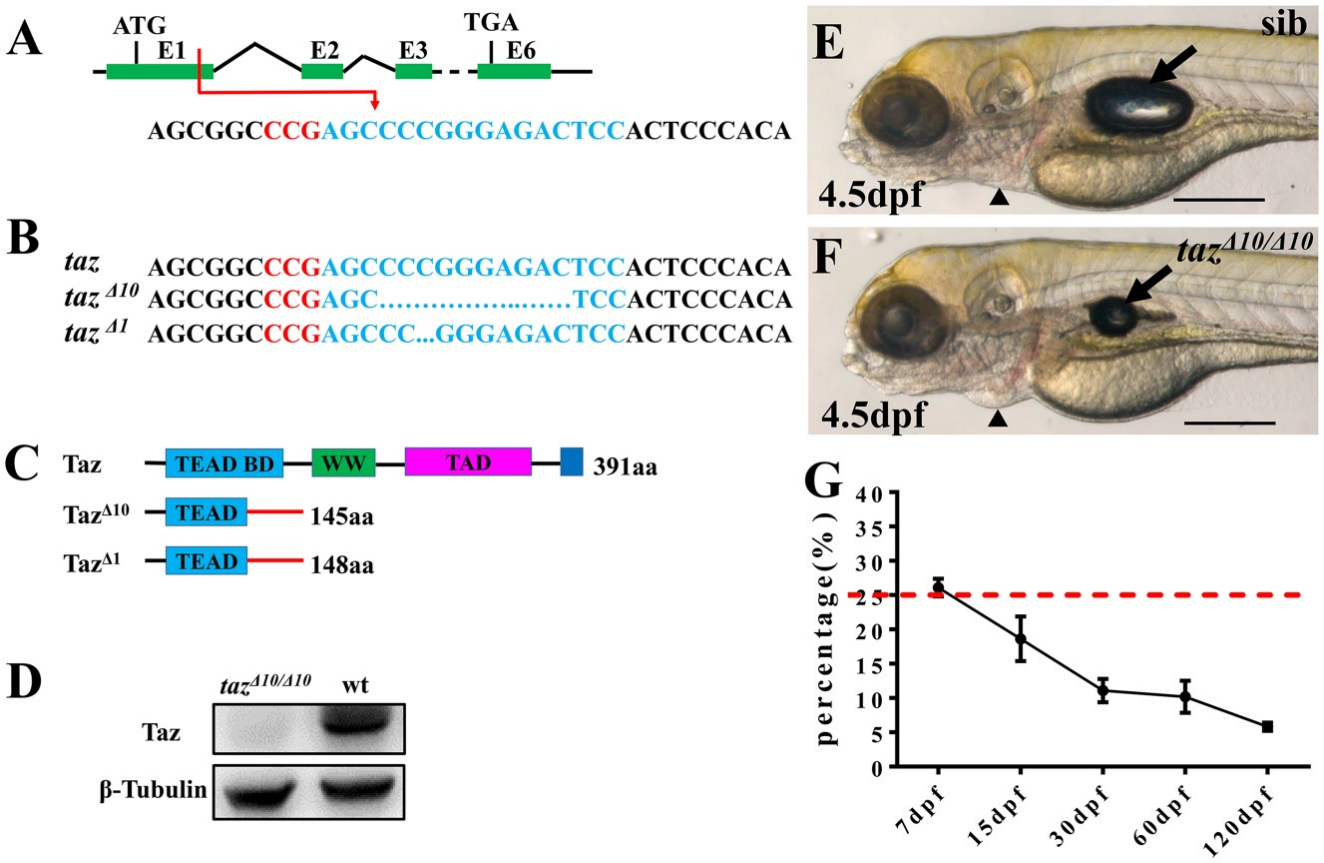
Some homozygous *taz* mutants can survive to adulthood. (**A-C**) Schematic of CRISPR/Cas9 knock-out of *taz*. The target site (shown in blue) is located in exon 1 of *taz*, and the PAM region is shown in red (A). Two mutant alleles with small deletions, *taz* ^*Δ10*^ and *taz* ^*Δ1*^, were obtained (B), both of which result in frame-shifts of the *taz* open reading frame and truncated proteins (C). aa, amino acid; TEAD BD, Tead transcription factor binding domain (light blue); WW, dual tryptophan motif (green); TAD, transactivation domain (purple); PDZ domain (blue). **(D)** Western blot analysis shows Taz protein in wild type but not in *taz*^*Δ10/ Δ10*^ mutant embryos. β-Tubulin is used as a loading control. (**E, F**) Similar to wild type embryos (E), *taz*^*Δ10/Δ10*^ embryos are largely normal at 4.5 dpf, except for a smaller inflated swim bladder and mild pericardial edema (F). (**G**) Survival curve of *taz*^*Δ10/Δ10*^ in heterozygote intercrosses from larval to adult stages (shown as % survivors, from two independent experiments). The red dashed line indicates the expected 25% homozygous mutants in accordance with Mendelian segregation. Number of larvae/adults at each stage: 7 dpf (59/228), 15 dpf (37/200), 30 dpf (20/182), 60 dpf (22/216) and 120 dpf (10/174). Black arrow, swim bladder; black arrowhead, pericardium. Scale bar, 500 μm.

**Fig 2:**
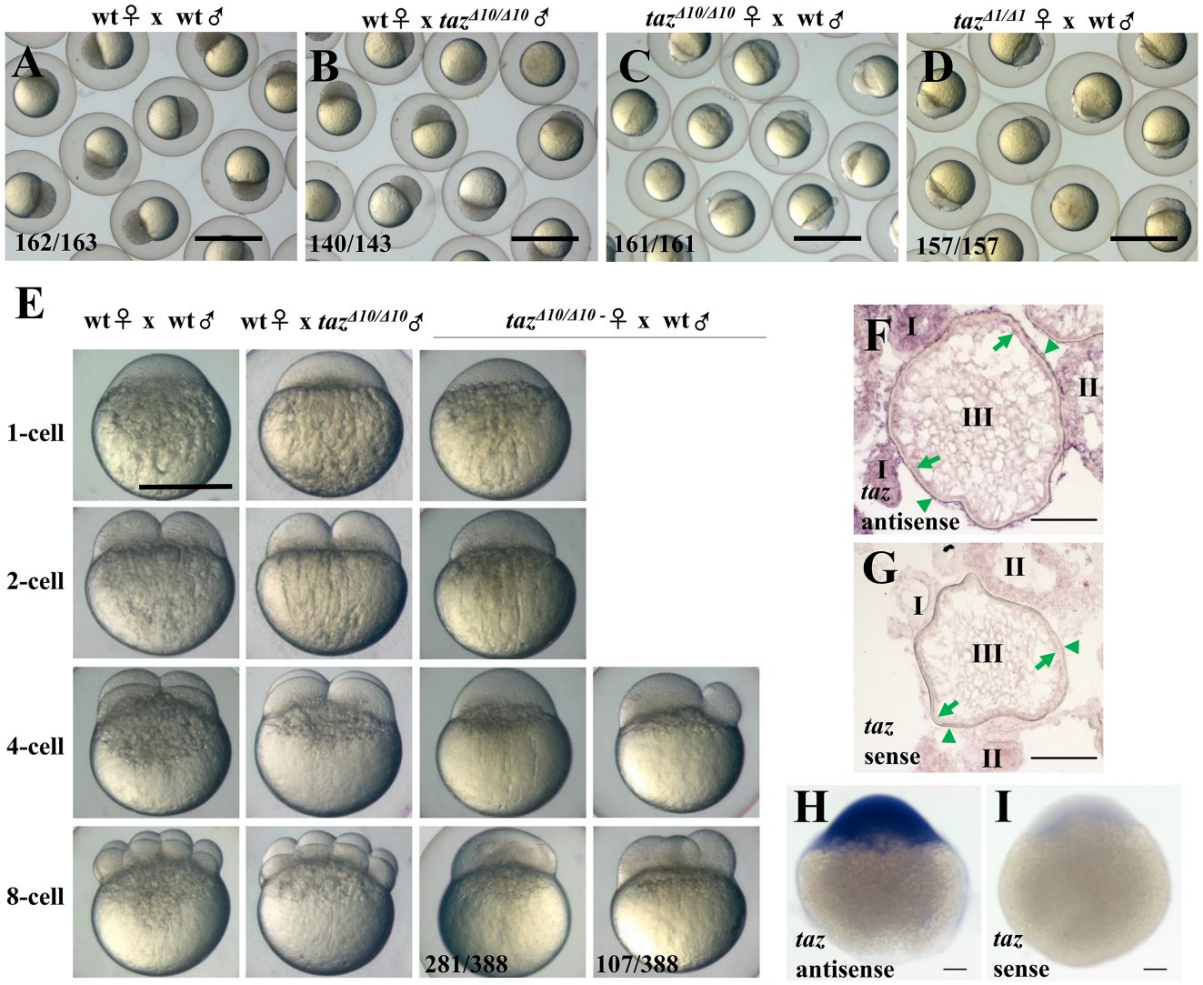
Loss of maternal Taz results in failure of eggs to develop. (**A-D**) 3 hpf embryos from crosses of a wild type female and male (A), wild type female with *taz*^*Δ10/Δ10*^ male (B), *taz*^*Δ10/Δ10*^ female with wild type male (C) and *taz*^*Δ1/Δ1*^ female with wild type male (D). Embryos from *taz*^*Δ10/Δ10*^ or *taz*^*Δ1/Δ1*^ females are arrested at the 1-cell stage. (**E**) Embryos from crosses of wild type female and male, wild type female with *taz*^*Δ10/Δ10*^ male, and *taz*^*Δ10/Δ10*^ female with wild type male are shown at 1-cell, 2-cell, 4-cell and 8-cell stages. Most embryos produced by *taz*^*Δ10/Δ10*^ females remain at the 1-cell stage, and a few initiate irregular cleavage planes at wild type 4-cell and 8-cell stage. (**F-I**) *In situ* hybridization to detect *taz* mRNA shows that *taz* transcripts are found in oocytes and follicle cells in ovary sections (F), and abundant expression in embryos at the 1-cell stage (H) when compared to sense probe control (I). Nonspecific signal is detectable in oocytes at stage I and II while using sense probe as control (G). Oocytes at stages I, II, III are shown; green arrow, the oocyte cortex; green arrowhead, follicle cells. Scale bar, 1 mm in A-D, 0.5 mm in E and 100 μm in F-I.

To study Taz function, we knocked out *taz* by targeting the first exon using CRISPR/Cas9 genome editing and recovered two mutant alleles, *taz*^*Δ10*^ and *taz*^*Δ1*^, both of which produce mutant transcripts that encode truncated proteins with 148 and 145 amino acids, respectively (Fig 1A, 1B and 1C). Importantly, no Taz protein was detected in *taz*^*Δ10/Δ10*^ mutant embryos (Fig 1D), suggesting that the lesion in *taz*^*Δ10/Δ10*^ results in a null mutant. Consistent with a previous report [32], *taz*^*Δ10/Δ10*^ mutant embryos displayed relatively normal morphology with exception of a smaller swim bladder than wild type and weak pericardial edema at 4.5 day post fertilization (dpf) (Fig 1E and 1F). However, it did not lead to embryonic lethality as indicated by the expected incidence of homozygous mutants from intercrosses of heterozygotes (~25% at 7 dpf), and in accordance with Mendelian segregation (Fig 1G). Some *taz*^*Δ10/Δ10*^ mutants could grow into adulthood, although the survival ratio was much lower than expected. Interestingly, while *taz*^*Δ10/Δ10*^ adult males were fertile (Fig 2B and 2E), all embryos from mating of *taz*^*Δ10/Δ10*^ adult females were arrested at the one-cell stage regardless of the genotype of the male (Fig 2C and 2E), and even though the females ovulated normally and produced eggs. We found similar phenotypes with the *taz*^*Δ1/Δ1*^ allele (Fig 2D), and all subsequent studies reported in this work were done using *taz*^*Δ10/Δ10*^ mutants. Since *taz*^*Δ10/Δ10*^ adult females are infertile, this suggests that *taz* is indispensable for producing normal eggs. Therefore, we surveyed if *taz* was expressed in oocytes. *In situ* hybridization showed that *taz* transcripts were found in the cortex of oocytes and the attached follicle cells (Fig 2F). Moreover, in one-cell stage embryos, where mRNAs are deposited in eggs by the mother, *taz* transcripts are abundant (Fig 2H), which is consistent with transcriptomic datasets [33] (http://www.ensembl.org/Danio_rerio/Location/View?r=22%3A38049130-38114599). Together, these data reveal that *taz*, a maternally expressed gene, is essential for fertilization.

### Taz function is not required for oogenesis or oocyte polarity

**Fig 3:**
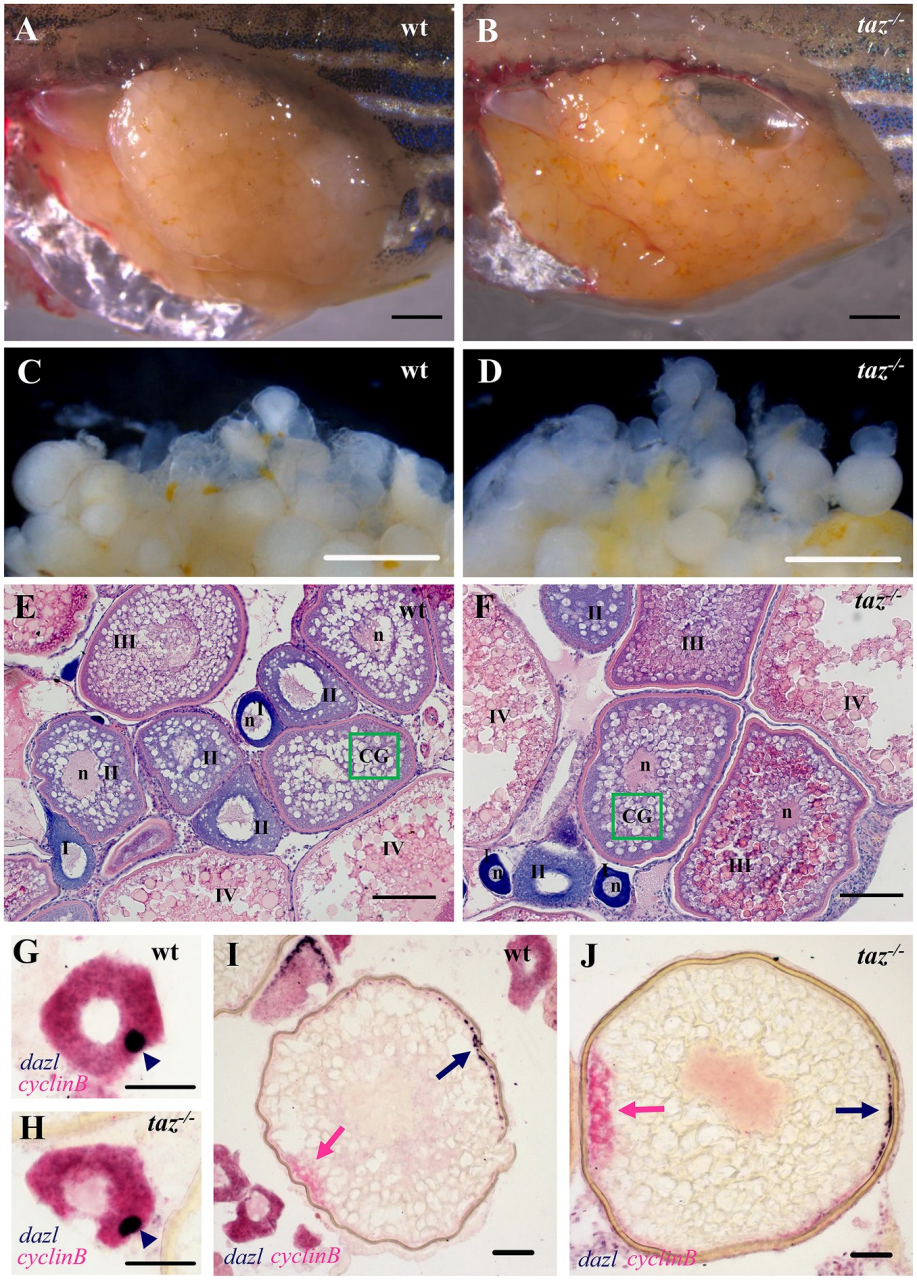
Oogenesis and oocyte polarity seems normal in *taz*^*-/-*^ zebrafish females. (**A-D**) Lateral views of dissected adult wild type and *taz*^*-/-*^ ovaries. Similar to wild type (A), the *taz* mutant ovary (B) is grossly normal, and *taz* mutant oocytes (D) are similar to wild type (C) in size and numbers. (**E-F**) Haematoxylin and eosin (HE) stained sections show no apparent morphological difference in oogenesis between wild type (E) and *taz*^*-/-*^ ovaries (F). Oocytes at stages I-IV are marked; n, nucleus; green box, CG (cortical granules). (**G-J**) *In situ* hybridization on sectioned ovaries to detect oocyte polarity markers show that animal-vegetal polarity is established in *taz*^*-/-*^ oocytes similar to wild type oocytes. The Balbiani body, and subsequently, vegetal pole labeled by expression of *dazl* in wild type and *taz*^*-/-*^ are normal in primary (G, H) and stage III (I, J) oocytes, and the animal pole, marked by *cyclinB* transcripts, is properly established in both wild type and *taz*^*-/-*^ oocytes (I, J). Dark blue arrowhead, Balbiani body; dark blue arrow, vegetal pole; pink arrow, animal pole. Scale bar, 1 mm in A-D, 100 μm in E-F, and 50 μm in G-J.

To determine the basis of the failure of *taz*^*Δ10/Δ10*^ eggs to progress beyond the one-cell stage, we examined the ovaries and oogenesis in mutant females. Compared with ovaries at the same stage in wild type females, the ovary of an 8-month old *taz* mutant female was grossly normal in the size, tissue composition and intraperitoneal position (Fig 3A and 3B), and there were no apparent morphological defects in the color, size and shape of oocytes (Fig 3C and 3D). Histological analysis showed that all stages of oocytes (stage I to IV) were present in *taz* mutant ovaries, and had no obviously difference from that in wild type controls (Fig 3E and 3F), indicating that the oogenesis was largely normal in *taz* mutants.

The establishment of animal-vegetal polarity in oocytes is a key event during oogenesis, and determines the formation of two major embryonic axes, the dorsal-ventral and left-right axis, in vertebrates [34]. Therefore, we examined if the failure of *taz* mutant oocytes to be fertilized was due to defects in animal-vegetal polarity. The Balbiani body (Bb) is the earliest vegetal structure in stage I oocytes, and can be marked by the expression of *dazl* transcripts [35]. In stage I oocytes of *taz* mutants, the Balbiani body appeared similarly as in wild type oocytes (Fig 3G and 3H). Furthermore, while *dazl* transcripts were found located in the vegetal pole, *cyclinB*, an animal pole marker, was distributed on the opposite side of oocytes in both *taz* mutant and wild type (Fig 3I and 3J), indicating that the animal-vegetal polarity was normally established in *taz* mutant oocyte. Similarly, expressions of other two polarity markers, *pou2* and *brl*, were not altered in mutant oocytes (S1 Fig). Taking together, we conclude that *taz* is not necessary for oogenesis or for the establishment of oocyte polarity in zebrafish.

### Taz is required for micropyle formation

**Fig 4:**
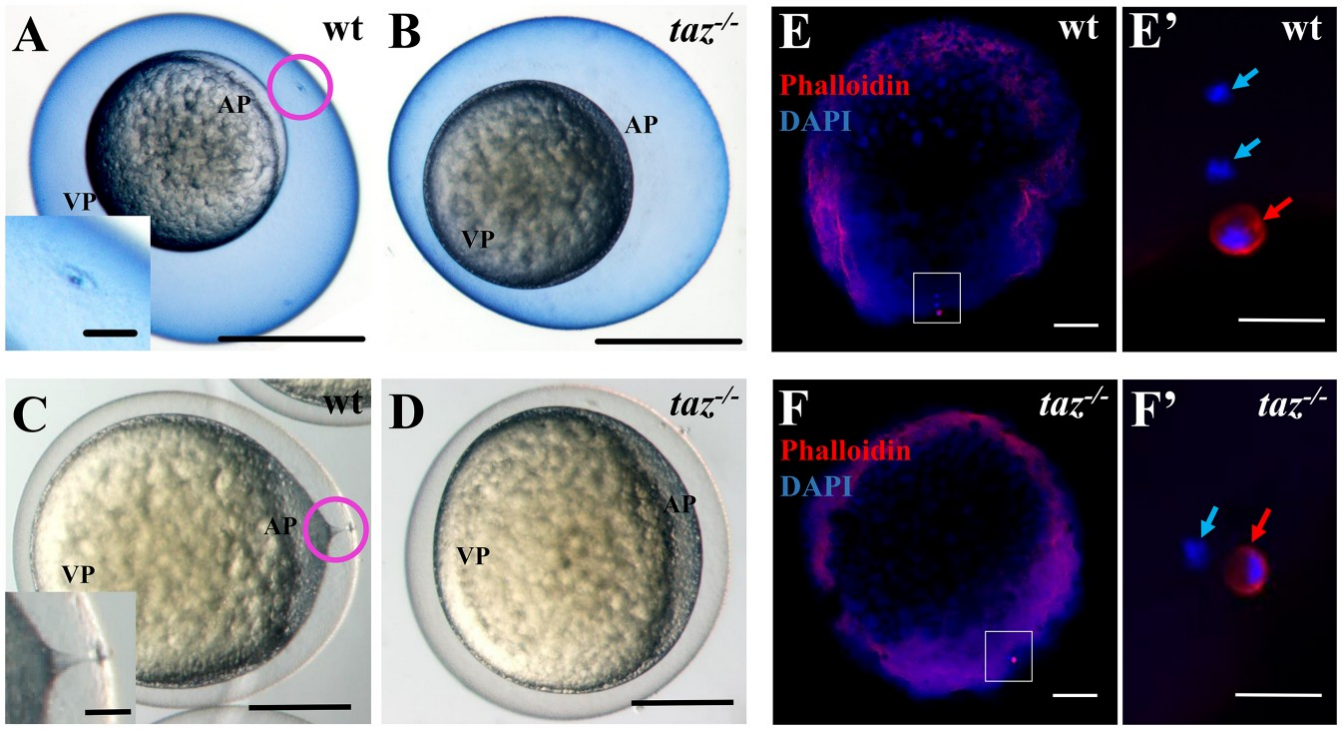
*taz* is essential for formation of the micropyle and fertilization. (**A-D**) At 15 minutes post fertilization, Coomassie Blue staining shows a single hole, the micropyle, in the chorion above the animal pole of wild type eggs (A, n = 92), but not in *taz*^*-/-*^ eggs (B, n = 131). Upon activation, a cytoplasmic extension towards the micropyle is observed in wild type eggs (C, n = 162). By contrast, no cytoplasmic projection is observed in *taz*^*-/-*^ eggs (D, n = 142). Insets in A and C are magnified images of the area within the pink circle. (**E-F’**) Two DAPI-stained pronuclei and one Actin-rich polar body are observed in a fertilized wild type eggs at 10 minutes post fertilization (E, E’, n = 61/63), whereas activated *taz*^*-/-*^ eggs show one pronucleus and one polar body (F, F’, n = 47/47), suggesting that *taz*^*-/-*^ egg is not fertilized, and the second polar body is extruded. White boxes in E to F denote regions magnified in E’ to F’. Red arrow, polar body; light blue arrow, pronucleus; AP, animal pole; VP, vegetal pole. Scale bar, 0.5 mm in A-B, 0.25 mm in C-D, 50 μm in insets in A and C, 100 μm in E-F and 20 μm in E’-F’.

Since oogenesis seemed normal in *taz* mutant ovaries, next we checked if fertilization of mutant eggs was normal. In teleost eggs, the micropyle is a narrow canal for sperm entry through the chorion during fertilization. While all wild type eggs had a single, animal-pole localized micropyle (Fig 4A), no micropyle was detected in *taz* mutant (Fig 4B). Furthermore, a single obvious cytoplasmic projection from the plasma membrane to the micropyle was present in wild type eggs shortly after egg activation (Fig 4C), whereas no protrusion was found in *taz* mutant eggs (Fig 4D). These observations strongly suggest that the lack of micropyle in *taz* mutant eggs results in their not being fertilized as sperm likely cannot enter the egg.

Once zebrafish eggs are activated, the second meiotic division is quickly completed, and the second polar body is extruded [36], a hallmark of the completion of the meiosis. To examine if the failure of fertilization of *taz* mutant eggs is due to no sperm entry, we performed DAPI and Phalloidin staining in activated eggs. While the pronucleus (from egg or sperm) is only stained by DAPI, the polar body from egg, surrounded by Actin, is detected by both DAPI and Phalloidin. After fertilization, sperm DNA enters the egg, and two pronuclei, from the egg and sperm, and one polar body were found in wild type eggs (Fig 4E and 4E’). However, in *taz* mutant eggs, a polar body and only one pronucleus were observed (Fig 4F and 4F’), indicating that meiosis is complete but there is a lack of sperm entry. These data demonstrate that oocyte meiosis can be completed without Taz, and the failure of fertilization in *taz* mutant egg is due to lack of the micropyle.

### Taz is specifically enriched in the micropylar cell

Our analysis suggests that oogenesis in *taz* mutant appears normal except for the lack of micropyle formation. In addition to oocytes, follicle cells are another group of cells that are essential for oogenesis to progress. In teleost eggs, follicle cells surround oocytes to provide nutrition. Some follicle cells specify into unique micropylar cells, which form one micropyle on each oocyte during stage III oogenesis in zebrafish [12, 37].

**Fig 5:**
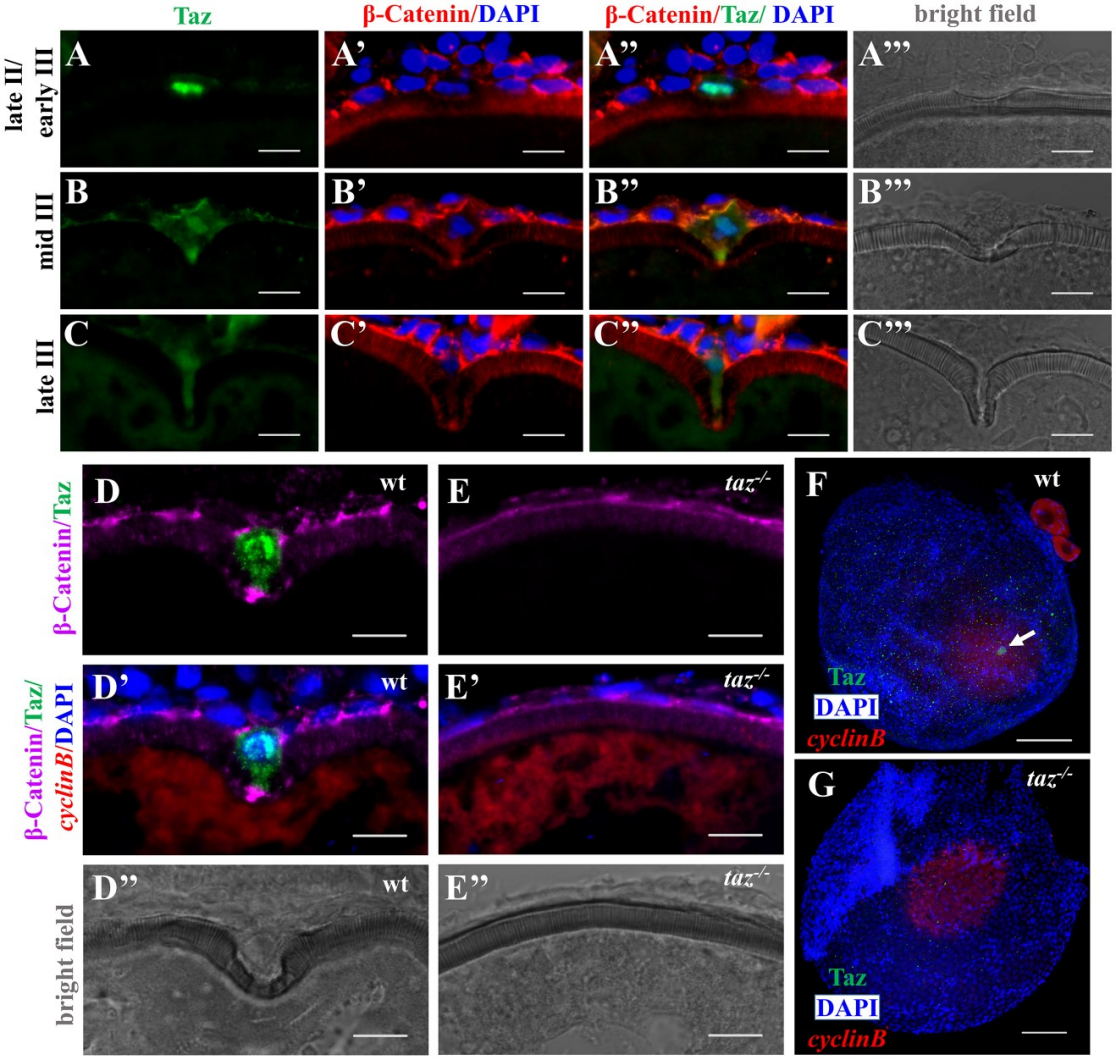
Taz is highly enriched in micropylar cells. (**A-C’”**) Immunofluorescence of Taz and β-Catenin in sectioned wild type oocytes at various stages: late II/ early III (A-A’”, n = 14), mid III (B-B’”, n = 14) and late III (C-C’”, n = 13). β-Catenin and DAPI label the cell membrane and nucleus, respectively. While basal levels are detected in follicle cells, in the micropylar cell high levels of Taz are detected, predominantly in the nucleus (A-C, A”-C”). Taz expression levels decrease as the micropylar cell develops (A-C, A”-C”). The micropylar cell undergoes dramatic shape change from flattened to ‘nail’-like to perforate the developing vitelline envelope (A’-C’, A”-C”). Bright field images show the gradual invagination of the vitelline envelope by the protruding micropylar cell (A’”-C’”). (**D-E”**) Immunofluorescence of Taz and β-Catenin in sectioned wild type and *taz*^*-/-*^ oocytes at stage III after *in situ* hybridization to detect *cyclinB*. In wild type ovaries, the high Taz expressing micropylar cell sits on the top of the oocyte animal pole, marked by *cyclinB*, and perforates the vitelline envelope (D-D”, n = 9). No Taz is detectable in *taz*^*-/-*^ oocyte (E), and neither the micropylar cell around the animal pole nor an invagination on the vitelline envelope is observed in mutant oocytes (E-E”, n = 11). (**F-G**) Immunofluorescence to detect Taz and *in situ* hybridization for *cyclinB* in wild type and *taz*^*-/-*^ oocytes at stage III. A single high Taz expressing micropylar cell is located at the animal pole of wild type oocyte (F, n = 13), but is not found in *taz*^*-/-*^ ovaries (G, n = 17). Two stage I oocytes, ubiquitously expressing *cyclinB*, are found adjacent to the stage III oocyte in figure F. White arrow, the micropylar cell indicated by high Taz expression. Scale bar, 100 μm in F and G and 10 μm in others.

To assess follicle cells during oogenesis, wild type and *taz* mutant ovaries were sectioned and stained with haematoxylin and eosin (HE). Compared with wild type, in *taz* mutant ovaries, follicle cells surrounding oocytes of all stages had no obvious difference in size, shape or numbers (Fig 3E and 3F). Interestingly, while follicle cells around oocytes showed basal levels of Taz expression, one particular cell was found highly enriched with Taz from late stage II to late stage III oogenesis (Fig 5A, 5B and 5C). This cell became larger than other follicle cells, and displayed a unique morphology change from flattened to ‘nail’-like shape (Fig 5A’, 5A”, 5B’, 5B”, 5C’ and 5C”). The micropylar cell depressed and eventually perforated the developing vitelline envelope (Fig 5A’”, 5B’” and 5C’”). Referring to morphological criteria, this Taz-enriched cell is the micropylar cell. Notably, Taz was predominantly distributed in the nucleus of micropylar cells, and the levels gradually decreased with progression of micropylar cell development (Fig 5A, 5B and 5C). The nuclear localization of Taz suggests that it might exert its function by transcriptional regulation of target genes. Moreover, high levels of Taz were found in the tip of cytoplasmic extension of the micropylar cell, especially in middle stage III oocytes (Fig 5B and 5B”). In sectioned wild type ovaries, the high Taz expressing micropylar cell is located on the top of the animal pole of oocyte marked by *cyclinB* (Fig 5D, 5D’ and 5D”). However, in *taz* mutant ovaries, neither the micropylar cell nor the invagination on the developing vitelline envelope was detected (Fig 5E, 5E’ and 5E”). We also performed Taz immunostaining in whole mount oocytes, and detected a single high Taz expressing micropylar cell on the top of animal pole in wild type stage III oocytes, but not in *taz* mutants (Fig 5F and 5G). These data suggest that Taz is required for the specification of micropylar cell, and the enrichment of Taz in micropylar cell agrees with an indispensable role for Taz in micropyle formation.

### Micropylar cells have bilobed nuclei

**Fig 6:**
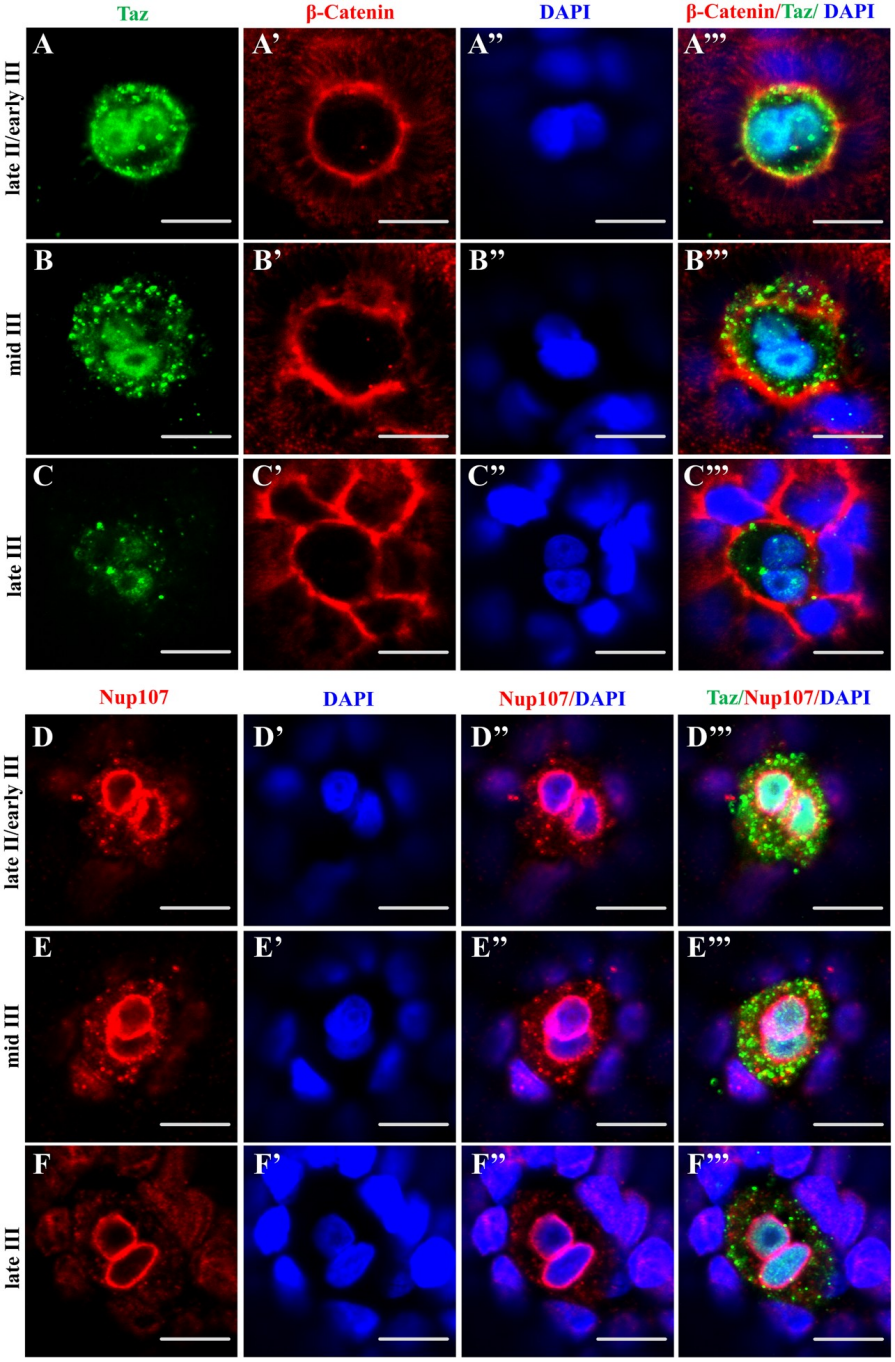
The nucleus in the micropylar cell is composed of two closely juxtaposed nuclei. (**A-C’”**) Single confocal plane of immunofluorescence of Taz and β-Catenin in whole mount wild type oocytes at three stages: late II/ early III (A-A’”), mid III (B-B’”) and late III (C-C’”) shows β-Catenin and DAPI in the cell membrane and nucleus respectively. The nucleus in most micropylar cells (57/60) is composed of two closely juxtaposed nuclei. (**D-F’”**) Similarly, single confocal planes of immunofluorescence of Taz and Nup107 (nuclear membrane marker) in stage late II/ early III (D-D’”), mid III (E-E’”) and late III (F-F’”) oocytes show two closely juxtaposed nuclei surrounded by continuous nuclear membranes in the micropylar cell (n = 32 oocytes). Scale bar, 10 μm.

Interestingly, in sections of wild type ovaries, the shape of the micropylar cell nucleus sometimes looked like two closely juxtaposed nuclei (Fig 5A). To examine the micropylar cell membrane and nucleus in detail, we performed co-immunostaining with Taz and β-Catenin in whole oocytes, while DAPI was used to label DNA. During oogenesis between late stage II to late stage III, two DAPI signals in close proximity within one cell are identified in almost all micropylar cell nuclei (Fig 6A–6C’”), being readily detected in late stage II/ early stage III oocytes, and gradually fading in late stage III oocytes. Co-labeling with an antibody towards Nup107, a nuclear pore marker, also showed two lumps of DAPI signals surrounded by a continuous nuclear membrane in micropylar cells from late stage II to late stage III oogenesis (Fig 6D–6F’”). However, we did not find clearly separated nuclei in all the oocytes (n = 132) that we assessed.

### Taz may regulate cytoskeletal dynamics in the micropylar cell

**Fig 7:**
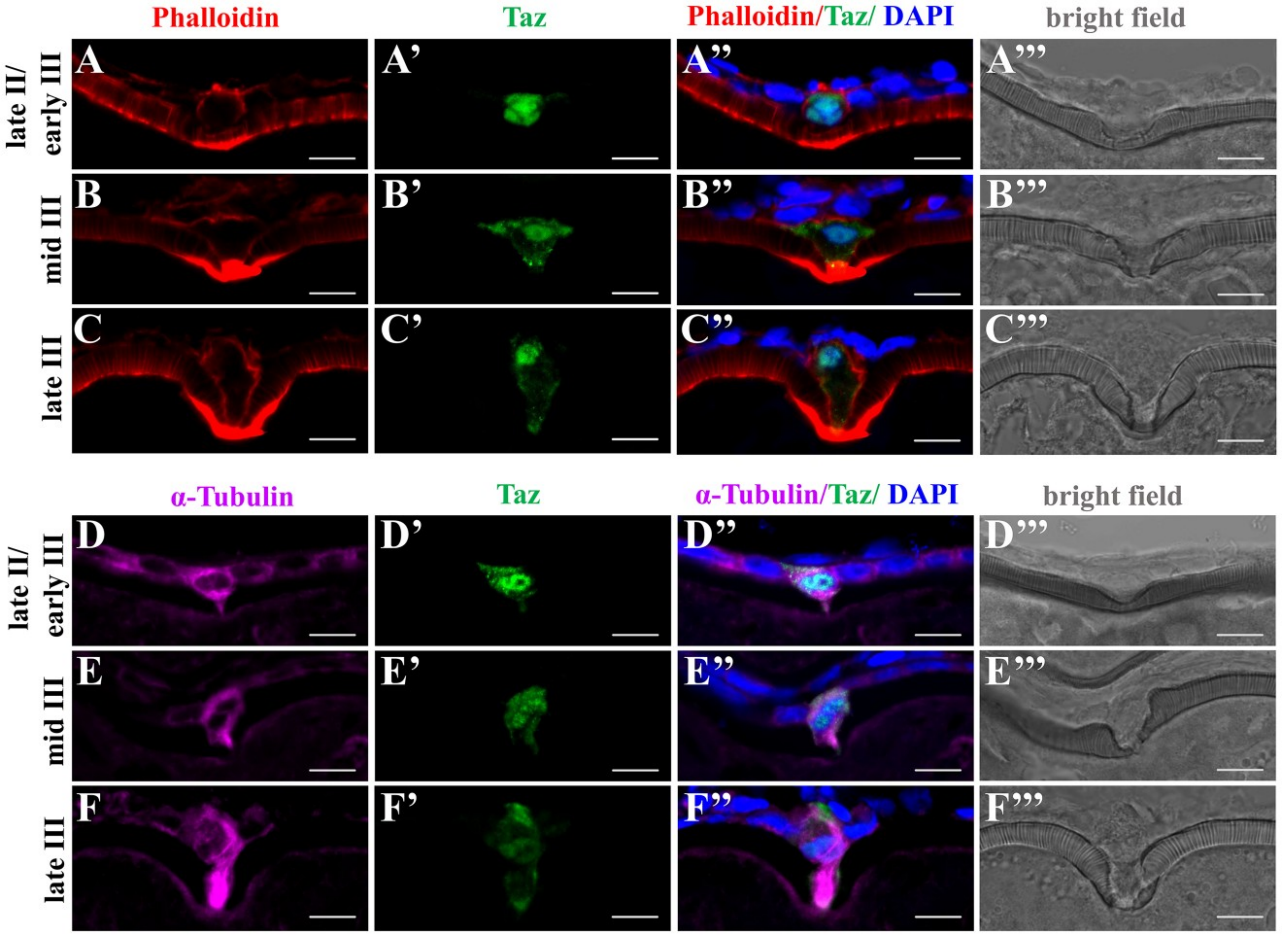
Taz may regulate cytoskeletal dynamics in the micropylar cell. (**A-F’”**) Immunofluorescence shows Taz and F-actin (A-C’”, n = 32) or α-Tubulin (D-F’”, n = 19) in sectioned ovaries; besides the invagination on the vitelline envelope, the expression of Taz and the shape of the micropylar cell changes with the growth of the micropylar cell; Phalloidin labelled F-actin bundles are found gradually deposited in the leading tip in the cytoplasmic extension of the micropylar cell and the part of oocyte cortex contacting the micropylar cell (A-C’”), and α-Tubulin is enriched in the cytoplasmic extension of the micropylar cell (D-F’”). Scale bar, 10 μm.

In many teleosts, formation of the micropyle is thought to require drilling of the vitelline envelope by the micropylar cell. During this process, the micropylar cell shape undergoes extensive changes [11, 13, 14], and the cytoskeleton might participate in this process. To assess the possible role of Taz in regulating cytoskeletal changes during micropyle formation, we performed co-staining of Taz with F-actin or α-Tubulin in wild type oocytes. We found that Actin filaments were enriched at the leading edge of oocyte cortex and the leading tip of micropylar cell, towards the indentation (Fig 7A). As oocytes mature, more Actin filaments were found deposited (Fig 7B and 7C, S2 Fig). Tubulin was also enriched in the cytoplasm of the micropylar cell, and in the cytoplasmic extension into the vitelline envelope (Fig 7D, 7E and 7F, S2 Fig). Considering the role of Yap1/Taz in regulating cytoskeleton in medaka and zebrafish [25, 27], the high expression of Taz and dynamic Actin and Tubulin in the micropylar cell suggests that Taz may regulate cytoskeletal arrangements during formation of a functional micropyle.

**Fig 8:**
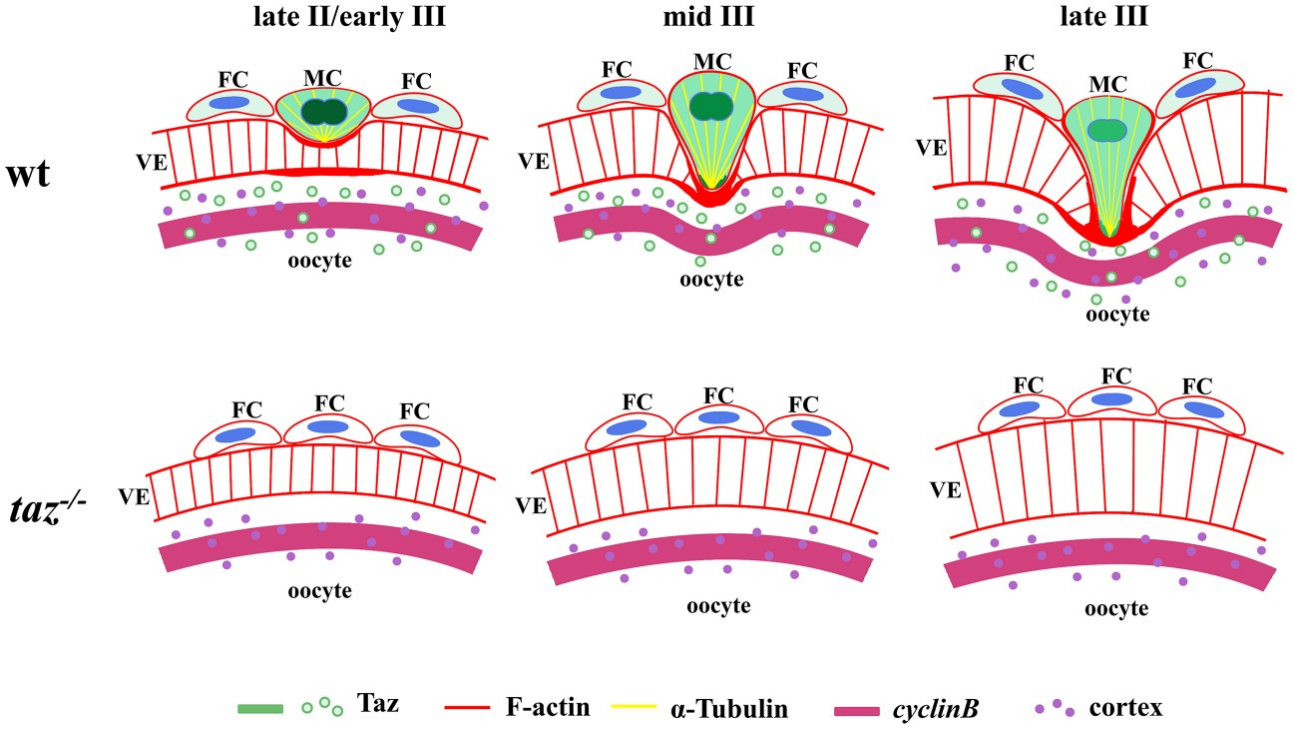
Model of micropylar cell specification in zebrafish. In late stage II/early stage III wild type oocytes, a particular follicle cell (FC) on the top of the animal pole, the micropylar cell (MC), expresses high level of Taz, predominantly in nucleus. Taz is expressed in FC and the oocyte cortex at a much lower level than in MC. The conical MC depresses the developing vitelline envelope (VE) and forms a shallow invagination. At middle and late stage III, the leading tip, enriched with Taz and F-actin, is formed in MC, and protrudes into the developing VE. Moreover, α-Tubulin accumulates in the cytoplasmic extension of MC, and concurrently, F-actin bundles deposit on oocyte cortex to form a leading edge. The dynamic cytoskeleton in MC and oocyte might facilitate perforation of MC into oocyte to form a micropyle. During MC development, the expression level of Taz gradually decreases, and the nucleus in MC, composed of two closely juxtaposed nuclei, becomes less clear. In *taz* mutants, Taz protein is not detected and no MC is formed, and this process likely does not happen.

Taken together, we have revealed a unique function of Taz in formation of the micropyle in zebrafish which is summarized in a model (Fig 8). In oocytes from late stage II to late stage III, the micropylar cell, sitting on the animal pole, becomes bigger and changes into a ‘nail’ shape. Taz is highly expressed in the micropylar cell. F-actin is deposited in the leading tip of the micropylar cell and the leading edge of oocyte cortex, and Tubulin is enriched in the micropylar cell cytoplasm and protrusion into the vitelline envelope. The dynamic cytoskeleton might facilitate perforation of the developing vitelline envelope. Without Taz, the micropylar cell is not specified, and no micropyle forms in *taz* mutant eggs.

## Discussion

The most interesting finding in this study is that mutations affecting Taz, a key effector of the Hippo signaling pathway lead to loss of a cell required for formation of the micropyle, the sperm entry port on eggs. Our findings identify the first molecular component in the establishment of this unique cell in zebrafish ovary. In *taz* mutant, loss function of Taz does not affect ovary and oocytes development, egg ovulation and the second meiosis of oocyte, but leads to failure of formation of the micropyle. Such a specific phenotype is due to the restricted high expression of Taz in the micropylar cell.

The high expression of Taz in one particular follicle cell at the animal pole in mid-oogenesis identifies Taz as the first molecular marker for the micropylar cell. With the aid of high Taz expression, the micropylar cell is easy to be identified. Besides the known characteristics, such as the big size and unique shape [12], most micropylar cells are found to have bilobed nuclei. These findings raise several interesting questions to be addressed in the future: Is DNA segregation incomplete in micropylar cells or are there two closely juxtaposed nuclei in micropylar cells? What leads to this: is this owing to incomplete cell division, cell fusion or proliferation? In preliminary experiments, we did not detect any pH3 signal, a maker for G2/M cell cycle phase, in the micropylar cell (S3 Fig), suggesting that cell proliferation probably does not underlie the bilobed nuclei. We also observed that there is a gradual down-regulation of Taz in the developing micropylar cell, with high expression levels of Taz in micropylar cells during middle oogenesis and lower levels from late stage III onwards. One possible explanation is that the signals that maintain Taz expression might be decreased as micropylar cell development progresses. It is also possible that Taz is not required when the micropylar cell becomes mature and finally degenerates.

Our study has demonstrated that Taz is required for micropylar cell specification from a follicle cell. At this stage, it is hard to distinguish if the high expression of Taz is a cause or consequence of micropylar cell specification. In a parallel study, Dingare *et*. *al*. demonstrate that Taz is required for micropylar formation in zebrafish, which agrees with our conclusion, and they also find that Taz is highly expressed in ectopic micropylar cells formed in *buc* mutant oocytes [38]. This suggests that once the micropylar cell is determined by other factors, it will express Taz. However, we cannot exclude the possibility that Taz is induced first. Identification of signals that induce Taz expression in follicle cells will help to address if Taz is a cause or consequence of micropylar cell specification. Besides upstream components in Hippo pathway which regulate Taz stability [21, 23], two aspects of oogenesis, which precede micropylar cell specification, need attention. One is the establishment of animal-vegetal polarity of the oocyte. It is widely accepted that follicle cells close to animal pole of oocyte contribute towards micropyle formation in many teleosts [14–16], suggesting that i) the animal pole determines the group of follicle cells competent for micropylar cell specification, and ii) animal pole specific-mRNAs could be inducers of Taz. The second is the growth of oocyte. Yap/Taz are known to act as mechanosensors [28, 29]. The volume expansion of oocytes may produce mechanical signals and activate Taz. These events, prior to micropylar cell specification, might induce Taz expression. Nonetheless, how a single follicle cell acquires micropylar cell fate is not clear. Inducible loss-of-function and overexpression of *taz* could address if Taz is a cause or consequence of micropylar cell specification. Lineage tracing in wild type and *taz* mutant ovaries, combined with single-cell gene expression profiling might also be informative [39–42].

Although we cannot identify if Taz is a cause or consequence of micropylar cell specification, our data reveal an essential role for Taz in this process. How does the micropylar cell exert its function by Taz expression? The most dramatic behavior of the micropylar cell is the shape change from a follicular epithelium into a highly polarized cell with a prominent projection, a process that is overtly similar to EMT in cancer. Besides EMT, Taz, as an oncoprotein, also promotes migration and invasion of human cancer cells, where cell shape changes are prevalent [24]. The micropylar cell is thought to bore through the developing vitelline envelope to form a channel, a process during which the cell shape must change greatly. Both cancer and micropylar cells are dynamic in shape, and therefore, it is reasonable to speculate that Taz works in a similar way in both processes. Yap/Taz regulates the cytoskeleton [25, 27], and nuclear localization of Taz in the micropylar cell may transcriptionally regulate Actin and Tubulin to drive morphogenesis of the micropylar cell, although the direct target downstream genes are unknown yet. In support of this possibility, a previous study in medaka showed that bundles of microtubules and tonofilaments are formed and elongated in the protruding cytoplasm of the micropylar cell during its penetration of the developing vitelline envelope [13]. In addition to its expression in the nucleus, Taz is also expressed in the cytoplasm of the micropylar cell, and enriched in the leading tip of cytoplasmic extension, where F-actin is extremely abundant. It is worthy of investigating if cytoplasmic Taz regulates the cytoskeleton, and the mechanism of regulation. In experiments to examine if the cytoskeleton is required for micropylar cell maintenance, Latrunculin B or Blebbistain was used to transiently inhibit Actin polymerization and Myosin II ATPase activity, respectively. Both inhibitors don’t have obvious effects on the morphology of micropylar cells (S4H” and S4I” Fig). However, dissociation of F-actin results in delocalization of Taz from nucleus to cytoplasm in the micropylar cell (S4H” Fig), while perturbation of Myosin II does not (S4I” Fig). These results are similar to observations in mammalian cell culture [43], indicating the cytoskeleton is required for maintenance of nuclear localization of Taz.

The first molecular evidence of regulation of micropyle formation comes from studies in a zebrafish mutant *bucky ball*, which have revealed that proper animal-vegetal polarity of the oocyte is essential for micropyle formation [19, 20]. In zebrafish *buc* mutant oocytes, the vegetal Balbiani body never forms, leading to an expansion of animal pole-specific gene expression (e.g. *vg1*) and multiple micropyles form in *buc* mutant eggs [19, 20]. Previous studies also found that extra territories of *vg1* transcripts coincide with the locations of ectopic micropylar cells in *buc* mutant oocytes [19]. By contrast, in *taz* mutant oocytes, animal-vegetal polarity is normal and yet, no micropyle forms. Therefore, the polarity of the oocyte alone is insufficient to determine micropyle formation, and additional mechanisms must govern micropyle cell fate. Our work identifies a new view of regulation during specification of this cell, and shows that follicle cells at the animal pole induce the formation of the micropyle in a Taz-dependent manner.

## Materials and methods

### Ethics statement

Zebrafish (*Danio rerio*) were raised and maintained in the fish facility in accordance with standard procedures [44] under approval from the Institutional Review Board of Southwest University (Chongqing, China).

### Zebrafish strains and embryos collection

AB^tü^ strain and subsequently generated *taz* mutant lines (*taz*^*Δ10/Δ10*^ and *taz*^*Δ1/Δ1*^) were used in this study. Embryos or oocytes were collected and staged as described [12, 45].

### Genomic DNA extraction

Embryos (or tail fin clips) were lysed in the lysis buffer (10 mM Tris pH 8.2, 50 mM KCl, 0.3% Tween-20, 0.3% Nonidet P40, 0.5 μg/μl Proteinase K (Fermentas)) at 55°C for 14 hours, followed by enzyme inactivation at 94°C for 20 minutes.

### Generation of *taz* mutants by CRISPR/Cas9 system

The target sequence of *taz* gRNA, 5’-GGAGTCTCCCGGGGCTCGG-3’ (PAM site underlined), was located in exon 1 of zebrafish *taz* gene. Zebrafish Cas9 mRNA and the *taz* gRNA were synthesized respectively according to the descriptions [46, 47]. After *ZCas9* mRNA (300 pg) and *taz* gRNA (50 pg) co-injection into one-cell stage wild type embryos, the lysate of 10 embryos at 24 hour post fertilization (hpf) was used as template for PCR with primers *taz* fw (5’-AGACCTGGACACGGATCTGGA-3’) and *taz* rv (5’-CACTGTATGCACTCCACTAACTGGT-3’). PCR products were sequenced to examine potential indels created in the *taz* gRNA target region. Embryos co-injected with functional *taz* gRNA and *ZCas9* mRNA were raised to adults (F0). F0 fish were screened to identify founders with progeny harboring the indels in *taz* gene previously found. Offsprings of identified F0 were raised. Individual F1 adults was reconfirmed by PCR using genomic DNA from tail fin clips, and indel types in fish were determined by sequencing.

### Genotyping of *taz* mutant

To detect *taz* Δ10 genotype, primers were designed to amplify specific bands by PCR with a common primer, *taz* fw2 (5’-CGATCGGACGCAGGAGGAACAA-3’), and two reverse primers, *taz* wt rv (5’-CGGGTGTGGGAGTGGAGTC-3’) and *taz* Δ10 rv (5’- CGGGTGTGGGAGTGGAGCT-3’). For *taz* Δ1 genotyping, the above *taz* fw and *taz* rv primers were utilized to obtain PCR products for sequencing.

### Western blot analysis

For preparation of zebrafish protein samples, 5 dpf embryos were homogenized in cold PBS with protease inhibitors (Roche) using syringe (1 ml) and needle (size 23G). The deyolked body fragments were collected and heated in whole cell lysis buffer (20 mM NaF, 1 mM DTT, 1 mM EDTA, 0.1 mM Na3VO3, 10% glycerol, 0.5% Nonidet P40, 280 mM KCl, 20 mM Hepes pH7.9) at 100°C for 10 minutes. Lysate supernatant was used for western blot analysis according to the standard protocol [48]. In this study, primary antibodies, anti-Taz (CST, 1:1000) and anti-β-Tubulin (Thermo, 1:1000) were used, while anti-mouse-IgG-HRP (Thermo, 1:5000) and anti-rabbit-IgG-HRP (Thermo, 1:5000) worked as secondary antibodies.

### Photograph of dissected ovaries

Adult females were euthanized by overdose tricaine treatment according to the guidelines of experimental animal welfare from ministry of science and technology of People’s Republic of China (2006), and abdominal tissue are removed by sharp scissors. Images were taken under a stereo microscope. Dissected ovaries were fixed in 4% PFA under room temperature for 2 hours, followed by images acquisition.

### Histology

Wild type and mutant ovaries were dissected from 8 month old females, and fixed overnight in saturated picric acid at room temperature. Fixed tissues were embedded in paraffin and sections were collected at 5-μm thickness using a microtome (Leica). Haematoxylin and eosin staining was performed according to standard protocol.

### *In situ* hybridization

Ovaries were dissected from adult abdomen and fixed in 4% PFA at room temperature for 2 hours. After overnight immersion in 30% sucrose in PBS at 4°C, the tissues were embedded in O. C. T. compound (Sakura) and frozen in ethanol at -80°C. Frozen tissues were sectioned at 10-μm thickness using a Cryotome (Leica). Serial sections were used for *in situ* hybridization as described previously [49]. Antisense RNA probes *cyclinB* [37, 50] and *pou2* [37, 50], labeled by fluorescein and digoxigenin, respectively, were used for marking animal poles in oocytes, while digoxigenin labeled *dazl* [35, 51] and *brl* [52, 53] were used to indicate vegetal poles. Whole mount and section *in situ* hybridization were performed according to methods used in a previous report to examine gene expression patterns of *taz* [54].

### Micropyle staining with Coomassie Brilliant Blue R

A nonspecific protein-staining dye, 2% Coomassie Brilliant Blue R (CB), was dissolved in DMSO. Prior to staining, the stock buffer was diluted in PBS (1:10). Eggs were collected in 5 minutes after activation, stained for 3 minutes, and rinsed thoroughly in PBS [55]. Stained eggs were examined and photographed under a stereo microscope.

### Oocyte activation and *in vitro* fertilization

For oocyte activation, Stage V oocytes were gently extruded from adult females, and activated by water. For *in vitro* fertilization, sperms were collected from adult males into Hank’s buffer and performed fertilization according to standard procedure [44].

### Isolation of oocytes from ovaries

For Immunohistochemistry, dissected ovaries were fixed in 4% PFA for 2 hours at room temperature, and oocytes were isolated in PBS by sharp forceps. For *in vitro* oocyte culture, dissected ovaries were gently dissociated by a Pasteur pipette in 90% L15 (pH9.0) medium (Gibco) with 0.5% BSA (Sigma).

### Inhibitors treatment in *in vitro* oocyte culture

Isolated oocytes were cultured in 90% L15 (pH9.0) medium with 0.5% BSA and 2ug/ml 17α-DHP (Sigma) at 28.5°C for 8 hours. Latrunculin B (Santa Cruz) or (-)-Blebbistain (MedChem Express), dissolved in DMSO, were supplemented into culture medium to final concentrations at 7.6 μM and 600 μM to inhibit Actin polymerization and Myosin II ATPase activity respectively.

### Immunohistochemistry

The frozen sections of ovaries were prepared as mentioned above. Ovary sections and oocytes were performed for immunohistochemistry as described previously [49]. Anti-Taz (CST; 1:200), anti-β-Catenin (Sigma; 1:200), anti-Nup107 (BioLegend; 1:400) and anti-α-Tubulin (Sigma; 1:200) were used as primary antibodies, and subsequent visualization was achieved by the application of secondary antibodies Alexa Fluor 488, Alexa Fluor 555 and Alexa Fluor 647 (Life Technology; 1:400). A solution of 4% BSA in PBS was used for blocking and diluting antibodies. FITC-Phalloidin (Sigma, 1:200) was used to detect F-actin. In some experiments, the animal pole was first labeled by *in situ* hybridization using *cyclinB* probe, followed by immunohistochemistry according to standard procedure. Before covering with Vectashield (Vector lab), DAPI (Roche) was employed to stain the nuclei. Images were acquired on a Zeiss LSM700 confocal microscope. Brightness of green fluorescence was slightly digitally enhanced to clearly show cytoplasmic expression of Taz on sectioned late stage III oocytes (see Figs 5C, 7C’ and 7F’).

## Supporting information

S1 Fig Oocyte polarity is normal in *taz*^*-/-*^ zebrafish females. *In situ* hybridization on sectioned ovaries showed that transcripts of *pou2* (A-B) and *brl* (C-D) were normally located on the animal and vegetal pole, respectively, in both wild type and *taz*^*-/-*^ oocytes. Dark blue arrow, vegetal pole; pink arrow, animal pole. Scale bar, 50 μm.
(TIF)

S2 Fig Cytoskeleton in the micropylar cell. Consecutive confocal sections at 2.5 μm intervals showing immunofluorescence of Taz, F-actin and α-Tubulin in a stage III wild type oocyte (n = 17). (A-B) show the micropylar cell body in which Taz is mainly expressed in the nucleus and α-Tubulin is in the cytoplasm. (C-F) show the cytoplasmic extension of the micropylar cell; α-Tubulin is enriched in the cytoplasm and F-actin is deposited at the leading tip (F). Dashed white circle, the micropylar cell. Scale bar, 10 μm.
(TIF)

S3 Fig The micropylar cell is pH3 negative. Immunofluorescence of F-actin and pH3 in wild type oocytes. The pH3 signal was not found in the micropylar cell (n = 70 oocytes). Insets are high magnification images of the micropyle in the yellow boxed area. Yellow arrow, pH3 positive cell, Scale bar, 100 μm; insets, 20 μm.
(TIF)

S4 Fig Transient disturbance of actin or Myosin does not significantly affect morphology of the micropylar cell. **(A-C’”)** Immunofluorescence shows Taz and F-actin in sectioned stage III oocytes after Latrunculin B (B-B’”, n = 7) or Blebbistain (C-C’”, n = 6) treatment. DMSO is the control (A-A’”, n = 7). Transient inhibition of actin polymerization (Latrunculin B) or Myosin II ATPase activity (Blebbistain) does not remarkably affect morphology of the micropylar cell (B”-C”). However, Latrunculin B treatment leads to cytoplasmic retention of Taz in the micropylar cell (6/7, B”), while Blebbistain does not (0/6, C”). Scale bar, 10 μm.
(TIF)
