# Supplementary figures and images for: The effector of Hippo signaling, Taz, is required for formation of the micropyle and fertilization in zebrafish
Source: PLoS Genet. 2019 Jan 4;15(1):e1007408. doi: 10.1371/journal.pgen.1007408 (PMC6334976; doi:10.1371/journal.pgen.1007408)

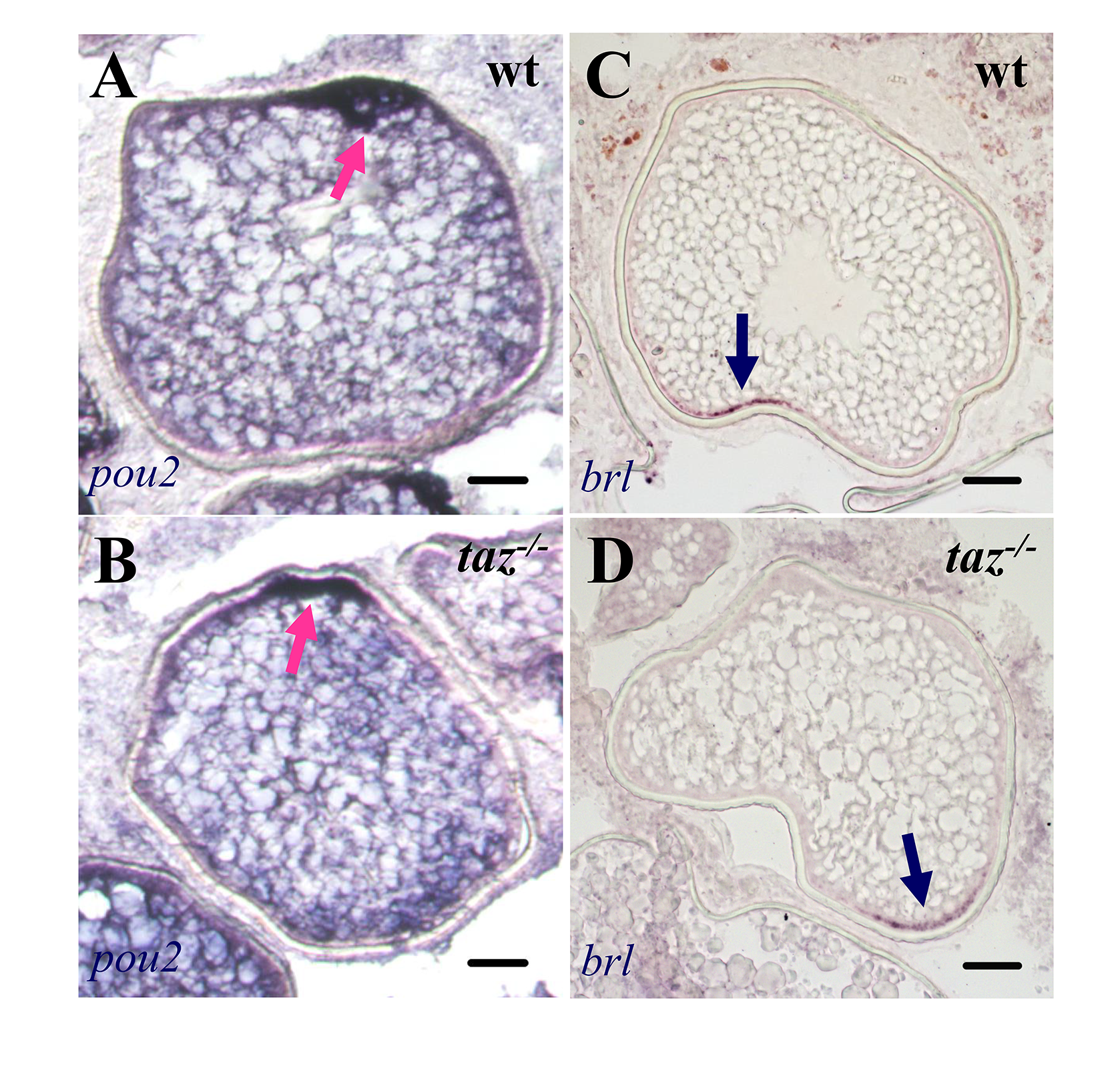

Supplement: S1 Fig — In situ hybridization on sectioned ovaries showed that transcripts of pou2 (A-B) and brl (C-D) were normally located on the animal and vegetal pole, respectively, in both wild type and taz-/- oocytes. Dark blue arrow, vegetal pole; pink arrow, animal pole. Scale bar, 50 μm. (TIF) [file pgen.1007408.s001.tif]

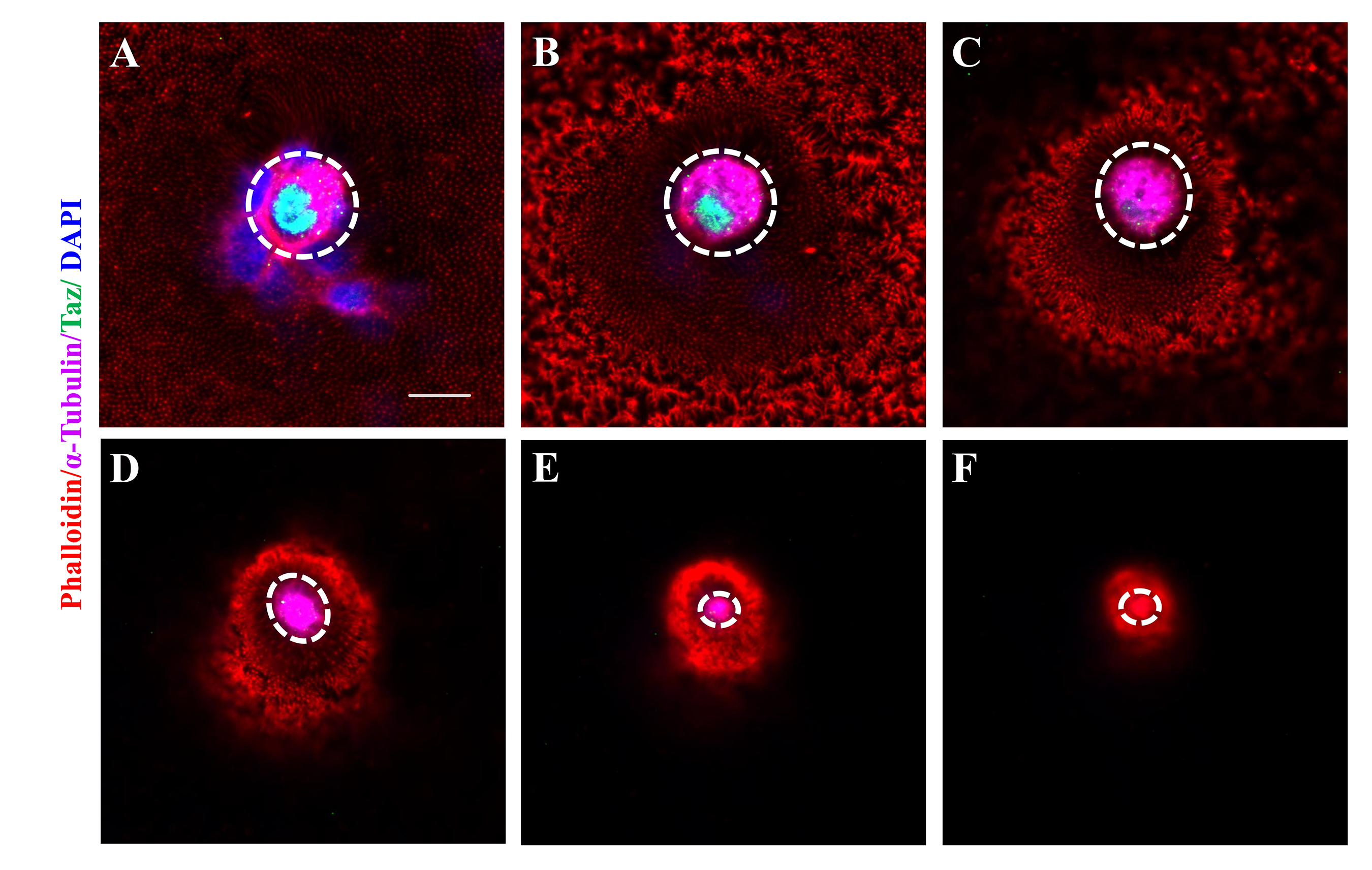

Supplement: S2 Fig — Consecutive confocal sections at 2.5 μm intervals showing immunofluorescence of Taz, F-actin and α-Tubulin in a stage III wild type oocyte (n = 17). (A-B) show the micropylar cell body in which Taz is mainly expressed in the nucleus and α-Tubulin is in the cytoplasm. (C-F) show the cytoplasmic extension of the micropylar cell; α-Tubulin is enriched in the cytoplasm and F-actin is deposited at the leading tip (F). Dashed white circle, the micropylar cell. Scale bar, 10 μm. (TIF) [file pgen.1007408.s002.tif]

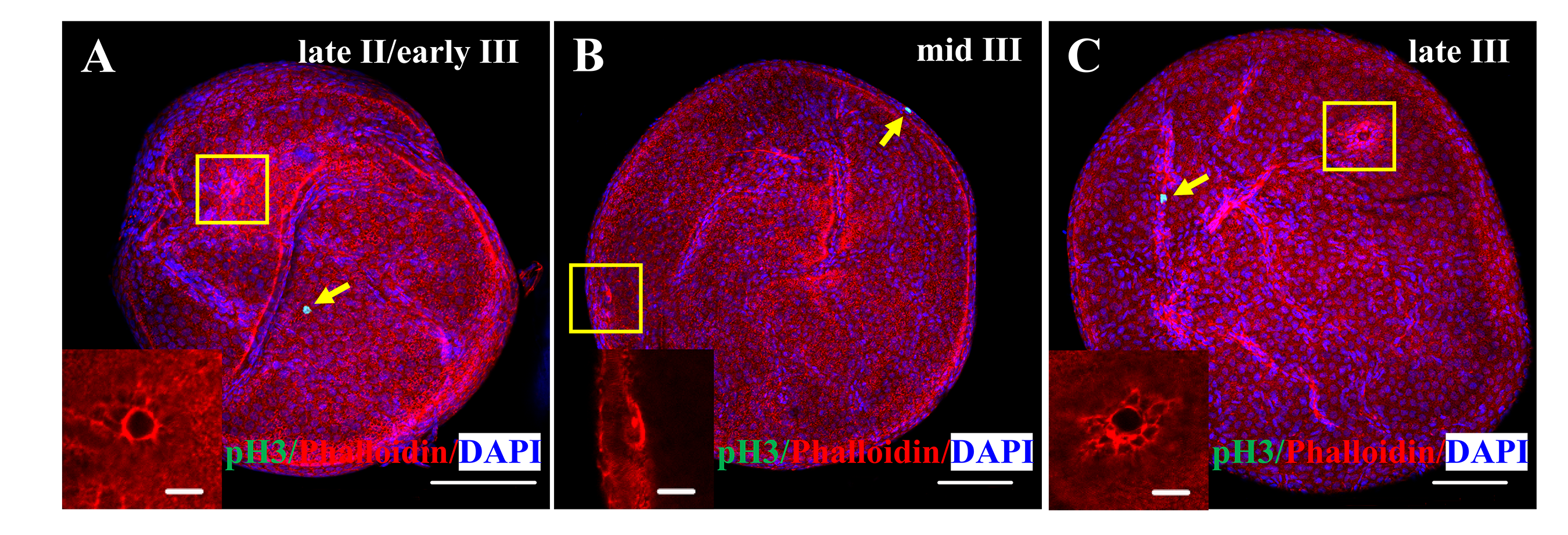

Supplement: S3 Fig — Immunofluorescence of F-actin and pH3 in wild type oocytes. The pH3 signal was not found in the micropylar cell (n = 70 oocytes). Insets are high magnification images of the micropyle in the yellow boxed area. Yellow arrow, pH3 positive cell, Scale bar, 100 μm; insets, 20 μm. (TIF) [file pgen.1007408.s003.tif]

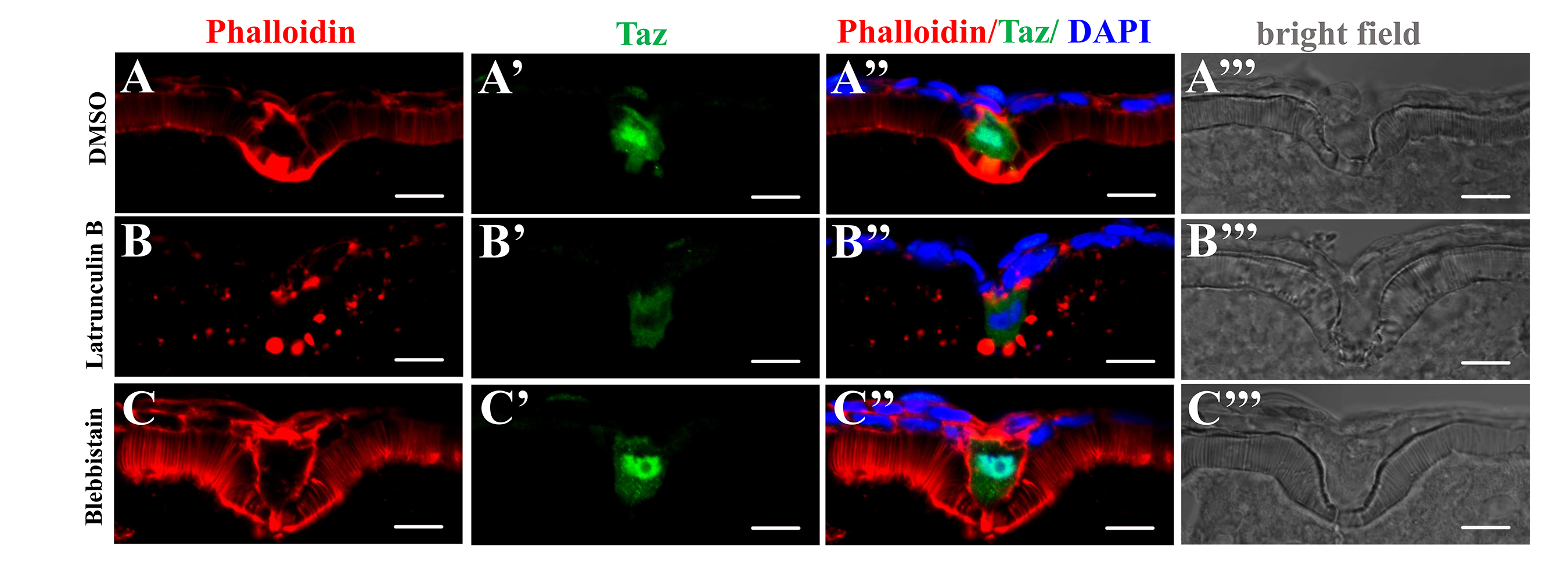

Supplement: S4 Fig — (A-C’”) Immunofluorescence shows Taz and F-actin in sectioned stage III oocytes after Latrunculin B (B-B’”, n = 7) or Blebbistain (C-C’”, n = 6) treatment. DMSO is the control (A-A’”, n = 7). Transient inhibition of actin polymerization (Latrunculin B) or Myosin II ATPase activity (Blebbistain) does not remarkably affect morphology of the micropylar cell (B”-C”). However, Latrunculin B treatment leads to cytoplasmic retention of Taz in the micropylar cell (6/7, B”), while Blebbistain does not (0/6, C”). Scale bar, 10 μm. (TIF) [file pgen.1007408.s004.tif]
